# Prevention of Ulcerative Colitis in Mice by Sweet Tea (*Lithocarpus litseifolius*) via the Regulation of Gut Microbiota and Butyric-Acid-Mediated Anti-Inflammatory Signaling

**DOI:** 10.3390/nu14112208

**Published:** 2022-05-26

**Authors:** Xiao-Qin He, Dan Liu, Hong-Yan Liu, Ding-Tao Wu, Hua-Bin Li, Xin-Shang Zhang, Ren-You Gan

**Affiliations:** 1 Research Center for Plants and Human Health, Institute of Urban Agriculture, Chinese Academy of Agricultural Sciences, Chengdu National Agricultural Science & Technology Center, Chengdu 610213, China; 18883394393@163.com (X.-Q.H.); liudan1534@163.com (D.L.); liuhongyan01@caas.cn (H.-Y.L.); 2 Key Laboratory of Coarse Cereal Processing (Ministry of Agriculture and Rural Affairs), Sichuan Engineering & Technology Research Center of Coarse Cereal Industralization, School of Food and Biological Engineering, Chengdu University, Chengdu 610106, China; wudingtao@cdu.edu.cn; 3 Guangdong Provincial Key Laboratory of Food, Nutrition and Health, Department of Nutrition, School of Public Health, Sun Yat-Sen University, Guangzhou 510080, China; lihuabin@mail.sysu.edu.cn; 4 Institute of Laboratory Animal Sciences, Academy of Medical Sciences & Sichuan Provincial People’s Hospital, Chengdu 610212, China

**Keywords:** inflammatory bowel disease, sweet tea, gut microbiota, SCFA–GPCR signaling cascade, HDAC3/NF-κB signaling pathway

## Abstract

Sweet tea (*Lithocarpus litseifolius* [Hance] Chun) is a new resource for food raw materials, with plenty of health functions. This study aimed to investigate the preventive effect and potential mechanism of sweet tea extract (STE) against ulcerative colitis (UC). Briefly, BABL/c mice were treated with STE (100 and 400 mg/kg) for 2 weeks to prevent 3% dextran sulfate sodium (DSS)-induced UC. It was found that STE supplementation significantly prevented DSS-induced UC symptoms; suppressed the levels of pro-inflammatory mediators, such as myeloperoxidase and tumor necrosis factor-α; increased the levels of anti-inflammatory cytokines; and up-regulated the expression of tight junction proteins (Zonula occludens-1 and Occludin). STE also altered the gut microbiota profile of UC mice by increasing *Bacteroidetes*, *Lactobacillus*, *Akkermansia*, *Lachnospiraceae*_NK4A136_group, and *Alistipes* and inhibiting *Firmicutes*, *Proteobacteria*, and *Helicobacter*, accompanied by a significant increase in the content of butyric acid. Moreover, STE increased the expression of G-protein-coupled receptor (GPR) 43 and GPR109A and inhibited the expression of histone deacetylase 3 (HDAC3) and nuclear factor-κB p65 (NF-κB p65) in the colon. In conclusion, this study indicated that STE has a good preventive effect on UC by regulating gut microbiota to activate butyrate-GPR-mediated anti-inflammatory signaling and simultaneously inhibit HDAC3/NF-κB inflammatory signaling.

## 1. Introduction

Ulcerative colitis (UC) is a recurrent inflammatory bowel disease with typical clinical symptoms, including bloody stools, diarrhea, and abdominal pain [1]. Epidemiological studies have shown that the incidence and prevalence of UC have increased worldwide since 1990 and may reach up to 505 and 58 cases per 100,000 people by 2025, respectively, indicating that UC has become a global disease threatening human health [2,3]. To date, although the etiology and pathogenesis of UC have not been completely elucidated, most research has confirmed that unhealthy dietary habits, environmental variables, host susceptibility genes, immune dysfunction, and gut microbiota disturbance are closely related to the occurrence of UC [4]. In particular, the intestinal barrier dysfunction caused by gut microbiota dysbiosis and early inflammatory response plays a critical role in the pathology of UC [1,5]. The conventional intervention drugs, including corticosteroids, 5-aminosalicylates, immunomodulators, and monoclonal antibodies, generally offer a limited curative effect and have a range of side effects [6]. Therefore, it is vital to explore safe and efficient methods to prevent or ameliorate UC.

In recent years, increasing attention has been directed to natural plant bioactive compounds, such as polyphenols and flavonoids, to prevent UC damage, with the advantage of powerful anti-inflammatory capacity and limited or no side effects [7,8]. *Lithocarpus litseifolius* (Hance) Chun, commonly known as sweet tea, is a new food resource with both medicinal and edible functions [9]. There is a long history of sweet tea being used as herbal tea or traditional medicine to prevent diabetes, hypertension, and hyperlipidemia [10]. Modern studies have indicated that those effects are mainly associated with its main bioactive dihydrochalcones, especially phlorizin, phloretin, and trilobatin [9,11]. Phloretin intervention was reported to be effective in inhibiting DSS-induced UC symptoms through its anti-inflammatory and antioxidant properties [12]. In addition, phlorizin was proved to have a positive effect on regulating the imbalance of intestinal flora and repairing the integrity of the intestinal barrier [13]. Furthermore, trilobatin of sweet tea could inhibit the mRNA expression of tumor necrosis factor-alpha (TNF-α), interleukin (IL)-1β, and IL-6 induced by lipopolysaccharide (LPS) [14]. The above studies indicate that dihydrochalcones of sweet tea may exhibit significantly therapeutic potential against UC. Moreover, pharmacokinetic studies have manifested that a large amount of poorly absorbed dihydrochalcones is accumulated in the intestine system and used by gut microbiota, suggesting that intestinal microbes and their metabolites, such as short-chain fatty acids (SCFAs), may play an essential role in dihydrochalcone-mediated alleviation of colon inflammation [5,13]. However, the anti-UC effects and related molecular mechanism of sweet tea remain unclear. Therefore, the preventive effect of sweet tea extract (STE) on UC animal models and its possible mechanisms in terms of restoring the intestinal barrier, inhibiting inflammation, and regulating gut microbiota were intensively explored in this study.

## 2. Materials and Methods

### 2.1. Materials and Reagents

Sweet tea was collected from Sichuan Mu Jiang Ye Ke Tea Co., Ltd. (Chengdu, China). Dextran sulfate sodium salt (DSS, molecular weight 36–50 kDa); salicylazosulfapyridine (SASP); and standards for SCFAs, including acetic acid, propanoic acid, isobutyric acid, butyric acid, isovaleric acid, valeric acid, caproic acid, and isohexanoic acid, were purchased from Sigma-Aldrich (St. Louis, MO, USA). The commercial kits for myeloperoxidase (MPO), glutathione (GSH), malondialdehyde (MDA), superoxide dismutase (SOD), TNF-α, transforming growth factor-β (TGF-β), IL-10, IL-6, IL-1β, LPS, and urine fecal occult blood were obtained from Nanjing Jiancheng Bioengineering Co., Ltd. (Nanjing, China). The rabbit polyclonal antibodies specific to Zonula occludens-1 (ZO-1), Occludin, G-protein-coupled receptor 43 (GPR43), G-protein-coupled receptor 109A (GPR109A), histone deacetylase 3 (HDAC3), and NF-κB p65 were obtained from Abcam (Shanghai) Trading Co., Ltd. (Shanghai, China).

### 2.2. Preparation and Compositional Analysis of STE

The preparation of STE was based on our previous study [15]. Briefly, the dried sweet tea sample (1.0 g) was mixed with 20 mL of 70% ethanol solution and extracted by a computer-based microwave-ultrasonic-ultraviolet multifunctional catalytic synthesizer (XH-300UP, Beijing, China) at the power of 450 W at 25 °C for 20 min. Then the mixture was centrifuged at 4200× *g* for 8 min, and the supernatant was collected and concentrated at 45 °C under vacuum and then freeze-dried to obtain STE. The total phenolic content (TPC) was determined by the Folin–Ciocalteu assay in gallic acid equivalents (GAEs), and the total flavonoid content (TFC) was determined as described previously [10]. In addition, the main phytochemical components of STE were identified and quantified by high-performance liquid chromatography (HPLC) analysis equipped with an Agilent Zorbax SB-C18 column (4.6 × 150 mm, 5 µm) by comparing the retention time with standards [15]. The mobile phase for HPLC was composed of water containing 0.1% formic acid (solvent A) and acetonitrile (solvent B). In addition, the elution program was carried out as follows: 5–25% solvent B for 0–10 min, 25–30% solvent B for 10–15 min, 30–95% solvent B for 15–20 min, and 95–5% solvent B for 20–25 min. The column temperature, the injection volume, the flow rate, and the detection wavelength were set at 40 °C, 10 µL, 1 mL/min, and 280 nm, respectively.

### 2.3. Animal Experiments

Fifty specific pathogen-free grade male BABL/c mice (6–7 weeks old) obtained from the Laboratory Animal Research Institute of Sichuan Provincial People’s Hospital (Chengdu, China) were housed five per cage in a specific pathogen barrier facility with controlled light (12 h/12 h light/dark cycle), temperature (25 ± 3 °C), and humidity (50 ± 10%). In addition, standard food and water were provided ad libitum throughout the experiment. All animal experiments were approved by the ethical committee of the Institute of Laboratory Animal Sciences, Sichuan Academy of Medical Sciences, and Sichuan Provincial People’s Hospital, with the permit number SYXK-2018-058.

**Figure 1:**
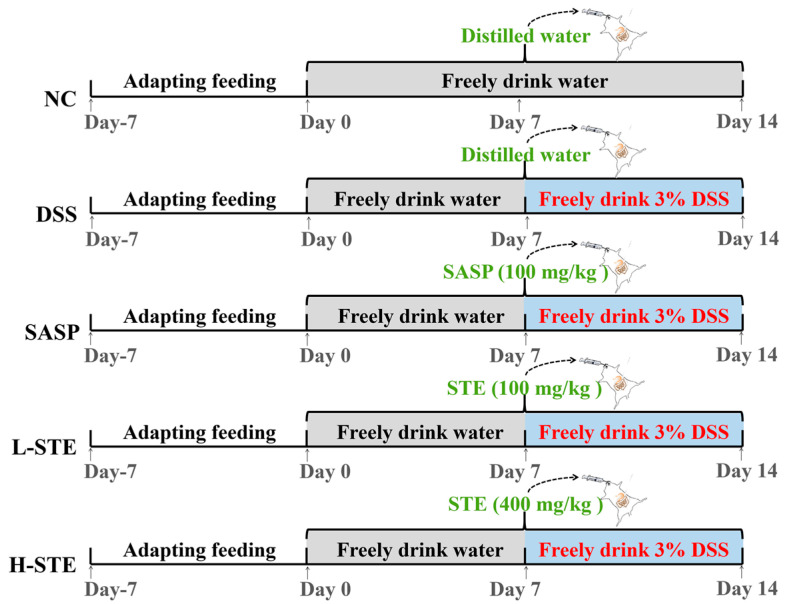
Schematic representation of the experimental design. NC, normal control group; MC, DSS-induced UC model group; SASP, suffasalarin intervention group at the dose of 100 mg/kg; L-STE, STE intervention group at the dose of 100 mg/kg; H-STE, STE intervention group at the dose of 400 mg/kg.

After 7 days of acclimatization, 50 mice were randomly assigned into the normal control group (NC), the DSS-induced UC model group (MC), the salicylazosulfapyridine (100 mg/kg) + DSS group (SASP), the low-dose sweet tea extract (100 mg/kg) + DSS group (L-STE), and the high-dose sweet tea extract (400 mg/kg) + DSS group (H-STE) (*n* = 10 per group). The mice in all groups except NC received 3% DSS in the drinking water from the 8th day to the 14th day to induce UC as described previously [16]. In addition, the mice in the SASP, L-STE, and H-STE groups were administered by gavage salicylazosulfapyridine (100 mg/kg), a low dose of STE (100 mg/kg), and a high dose of STE (400 mg/kg) for 14 days, respectively (Figure 1). The dosages of STE were selected based on references and preliminary experiments [12,16]. Meanwhile, mice in the NC group and the MC group received an equal volume of distilled water by gavage every day for 2 weeks (Figure 1). During the experiment, the body weight, fecal characteristics, and hematochezia degree of the mice were recorded at the same time each day and the disease index (DAI) was calculated as per the previously proposed method [12]. Fecal samples of each mouse were collected during the last week and stored at −80 °C for the analysis of SCFAs. At the end of the treatment, the mice were sacrificed by cervical dislocation after ether anesthesia and their colons were resected and measured.

### 2.4. Biochemical Analysis of the Colon Tissue

The colon segments were homogenized with pre-cooled PBS (*w*/*v*, 1:9) and then centrifuged at 4000× *g* for 20 min at 4 °C. The supernatant was collected for the determination of SOD, GSH, and MPO activities; MDA content; and IL-6, IL-10, IL-1β, TGF-β, and TNF-α levels as directed by the commercial kits.

### 2.5. Determination of LPS in the Serum

Blood was obtained by retro-orbital collection after the anesthesia, and the serum was collected after centrifugation (4000× *g*, 15 min, 4 °C). The content of LPS in the serum was measured with the corresponding commercial kit.

### 2.6. Histopathological Examination and Immunohistochemical Analysis

The distal colon specimens were gently flushed with cold PBS to remove non-tissue matters and immobilized in 10% neutral formalin for 48 h. Then, the colon specimens embedded in paraffin were cut into 4 μm thick sections using a Leica-2016 pathological microtome (Leica Instruments Co., Ltd., Berlin, Germany), followed by hematoxylin-eosin (H&E) and Alcian blue periodic acid Schiff (AB-PAS) staining after deparaffinization. The extent of the inflammation and histological scoring was assessed with reference to a previously reported method [12].

Immunohistochemical assessment was carried out to investigate the expression location and strength of target proteins Occludin, ZO-1, GPR43, and GPR109A. Specifically, the deparaffinized sections of the colon were incubated with 3% methanol hydrogen peroxide at 25 °C for 10 min. Then the sections were immersed in 0.01 M citrate buffer solution (pH 6.0) and heated at 100 °C for antigen recovery. After the sections were allowed to naturally cool and washed with PBS three times, the blocking solution using goat serum was added and the sections incubated at 25 °C for 20 min. Then the primary antibodies against Occludin, ZO-1, GPR43, and GPR109A were added and the sections allowed to incubate overnight at 4 °C, and the biotinylated secondary antibody was added at 37 °C for 30 min. Finally, 3,3′-diaminobenzidine was added for color rendering. The images were studied by a microscope (BA400Digital, Motic Group Co., Ltd., Chengdu, China).

### 2.7. Western Blot (WB) Analysis

WB analysis was conducted with reference to previous reports [17,18]. Briefly, the proteins in the colon tissue lysates were separated by 10% SDS-polyacrylamide gel and blotted on the polyvinylidene difluoride membranes. After being blocked by a 5% skim milk powder solution for 2 h, the membranes were incubated with the diluted primary antibodies against HDAC3 and NF-κB p65 (1:2000 dilution) at 4 °C overnight. Then, after being washed by diluted tris buffered saline with Tween 20 three times, the membranes were incubated with horseradish-peroxidase-conjugated secondary antibodies (1:5000 dilution) for 2 h. Finally, the immunoblots were visualized by the enhanced chemiluminescence reagent and quantified by ImageJ software.

### 2.8. Determination of SCFA Composition in Feces

Fecal samples (1.0 g) were mixed with 100 μL of phosphoric acid (15%, *v*/*v*), 200 μL of internal standard solution (isohexanoic acid, 125 μg/mL), and 400 μL of ether and left for 3 min and then centrifuged at 10,000× *g* at 4 °C for 15 min. The supernatant was obtained and SCFA composition was analyzed by Thermo TRACE 1310-ISQ Gas-Mass Spectrometer (Thermo Scientific, San Jose, CA, USA) according to the method reported previously [19].

### 2.9. 16S rRNA Gene Sequencing

Five mice from each group were randomly selected to collect cecal contents for gut microbiota analysis. The cecal contents were quickly frozen in liquid nitrogen and sent for 16S rRNA gene sequencing (BioNovoGene Technology Co., Ltd., Suzhou, China). Total bacterial genomic DNA was extracted from cecal contents using the QIAamp DNA Stool Mini Kit (Qiagen, Hilden, Germany) according to the instructions. Then, PCR amplification and sequencing of V3-V4 regions were performed on the Illumina NovaSeq system (Illumina, San Diego, CA, USA). The sequencing data were analyzed using the BioNovoGene Cloud Platform Quantitative Insights into Microbial Ecology (QIIME, V1.9.1).

### 2.10. Statistical Analysis

All experiments were conducted in parallel at least three times, and the data were presented as the mean ± the standard deviation (SD). The normality of the data was checked using the Kolmogorov–Smirnov test. One-way analysis of variance (ANOVA) and post hoc Duncan’s multiple test were used to analyze the data after the data passed the normality test by SPSS 20.0 software (SPSS Inc., Chicago, IL, USA), and statistical significance was defined at *p* < 0.05.

## 3. Results

### 3.1. Phytochemical Compounds of STE

**Figure 2:**
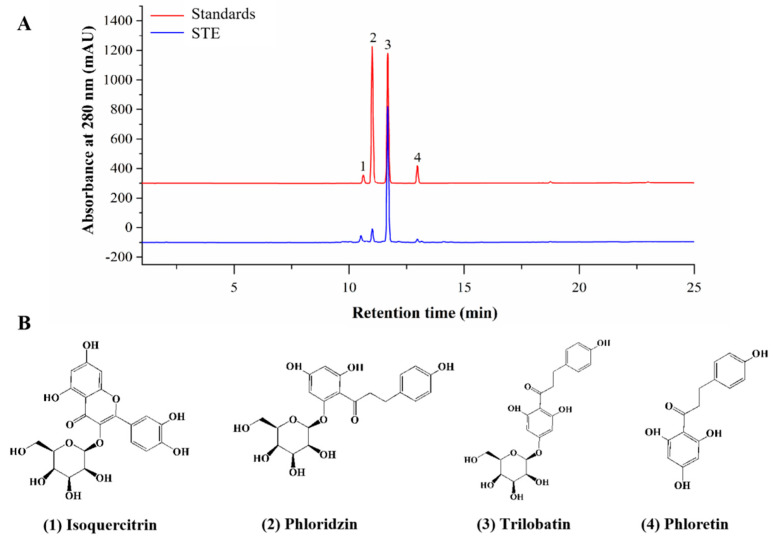
Phytochemical compounds of STE. (**A**) HPLC chromatograms of standards and sweet tea extract (STE) at 280 nm. The labeled number represents the compound: 1, isoquercitrin; 2, phloridzin; 3, trilobatin; and 4, phloretin. (**B**) The structures of isoquercitrin, phloridzin, trilobatin, and phloretin.

The TPC and TFC of STE were 200.68 ± 5.68 mg GAE/g DW and 38.95 ± 0.43 mg CE/g DW, respectively. In addition, the chromatograms of standards and STE and the identified components in STE are shown in Figure 2. A total of four active compounds were identified, of which trilobatin (215.23 ± 6.63 mg/g) showed the highest content, followed by phloridzin (48.99 ± 4.43 mg/g), phloretin (24.29 ± 3.43 mg/g), and isoquercitrin (10.22 ± 1.68 mg/g).

### 3.2. STE Prevented UC Symptoms

**Figure 3:**
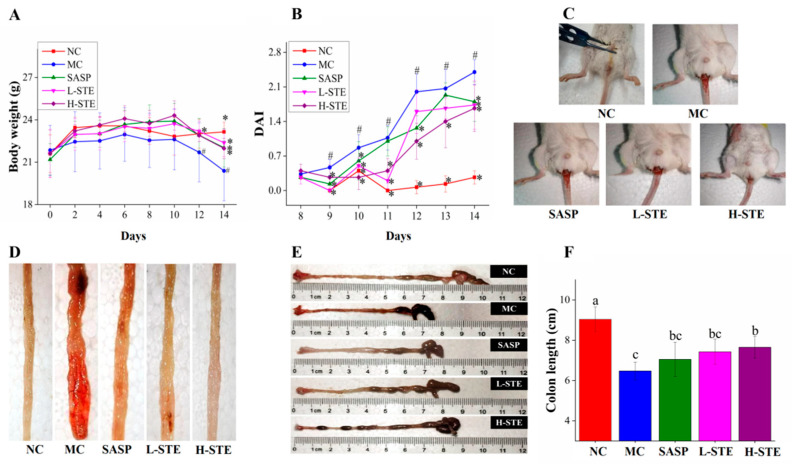
Effects of STE on disease symptoms of DSS-induced ulcerative colitis mice. (**A**) Body weight. (**B**) DAI scores during DSS treatment. (**C**) Typical images of hematochezia. (**D**) Typical images of colonic tissue bleeding. (**E**) Representative images of colon length. (**F**) Colon length. Values are expressed as the mean ± the SD (*n* = 10). # *p* < 0.05 compared to the NC group; * *p* < 0.05 and ** *p* < 0.01 compared to the MC group. Bars with different lowercase letters represent significant differences (*p* < 0.05).

As shown in Figure 3A, the administration of STE or SASP did not cause any UC symptoms in mice compared with the NC group and all mice had normal body weights during the first week. However, mice in the MC group exhibited weight loss gradually and their body weights were generally the lowest compared to other groups during the 2 weeks of intervention. In addition, the MC group mice exhibited diarrhea and hematochezia from day 10 (data not shown), accompanied by a significant increase in DAI and decrease in colon length (*p* < 0.05) (Figure 3C–F), indicating that the DSS-induced UC mouse models were successfully established. Importantly, STE administration markedly inhibited the increase in DAI and the shortening of the colon compared with the MC group, comparable to the SASP intervention (Figure 3C–F). These results demonstrate that STE could prevent the disease symptoms of UC.

### 3.3. STE Blocked Colon Histological Damage

**Figure 4:**
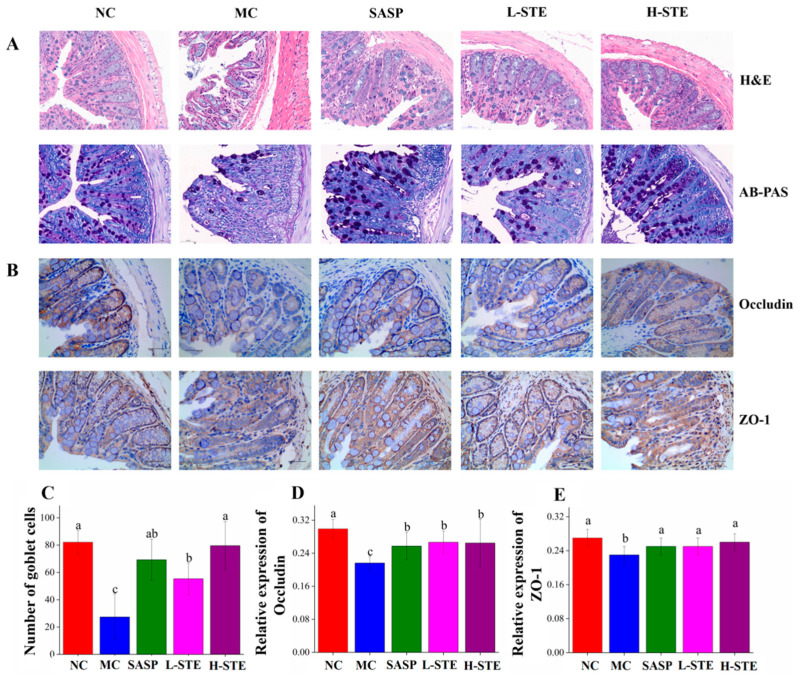
Effects of STE on colonic histopathological changes and tight junction protein expression in DSS-induced ulcerative colitis mice. (**A**) H&E staining and AB-PAS staining analysis of colon tissues (magnification of 400×). (**B**) The immunohistochemistry analysis of Occludin and ZO-1 in the colon tissue (magnification of 400×). (**C**) The goblet cell count on AB-PAS staining of colonic tissue. (**D**) The expression of Occludin in the colon. (**E**) The expression of ZO-1 in the colon. Values are expressed as the mean ± the SD (*n* = 5). Bars with different lowercase letters represent significant differences (*p* < 0.05).

We next analyzed whether STE could block the histopathological changes of the colon tissue. HE and AB-PAS (Figure 4A) staining of the NC group showed intact colon mucosal epithelia with normal numbers of goblet cells. On the contrary, the colon in the MC group had obvious mucosal structure damage, indicated by the large-scale degeneration and death of epithelial cells in the mucosal layer, different degrees of inflammatory cell infiltration, fibrous tissue proliferation, goblet cell depletion, and crypt loss (Figure 4A,C). However, compared with the MC group, mice orally administered STE effectively blocked these colon pathological injuries, as evidenced by the improved integrity of the mucosal layer, reduced cell infiltration, and increased numbers of goblet cells, generally in agreement with the SASP treatment (*p* < 0.05; Figure 4A,C).

In addition, since tight junctions play a central role in sustaining colonic mucosal barrier function [16], we further investigated whether STE could influence the main tight junction proteins Occludin and ZO-1 in the colon mucosal epithelia by immunohistochemical staining. As shown in Figure 4B, the positive staining of Occludin and ZO-1 was mainly on the surface of colonic epithelia and between the epithelial cells lining the crypt, while their expression levels in the MC group were significantly decreased compared to the NC group (*p* < 0.05; Figure 4D,E). On the contrary, both L-STE and H-STE intervention significantly blocked the loss of Occludin (Figure 4D) and ZO-1 (Figure 4E) compared to the MC group (*p* < 0.05) and their levels were generally equivalent to the SASP and NC groups. These results demonstrated that STE treatment could block the colon histological damage and epithelial barrier disruption by reducing tight junction protein loss and increasing goblet cell number in UC mice.

### 3.4. STE Diminished Inflammatory Mediators and Oxidative Stress

Inflammation and oxidative stress are two major factors that disrupt the intestinal barrier and promote the development of UC [12]. Therefore, we next investigated whether STE could inhibit inflammation and oxidative stress in UC mice. As shown in Table 1, the MC group mice manifested a notable increase in the levels of pro-inflammatory MPO, IL-6, IL-1β, TNF-α, and LPS, accompanied by a significant decrease in the contents of anti-inflammatory TGF-β and IL-10, compared to the NC group. In contrast, supplementation with STE overall restricted all pro-inflammatory mediators to the normal levels and simultaneously up-regulated the content of TGF-β and IL-10 (*p* < 0.05) compared to the MC group, in agreement with the effect of the positive control SASP.

In addition, compared to the NC group, DSS treatment also significantly inhibited the activities of SOD and GSH, accompanied by a significant increase in the oxidation end-product MDA (all *p* < 0.05) in the colon of the MC group (Table 1). On the contrary, STE intervention effectively prevented these changes (*p* < 0.05) and H-STE overall exhibited better in vivo antioxidant effects than the L-STE and SASP groups (Table 1). Collectively, these data suggest that STE could efficiently diminish DSS-induced inflammation and oxidative stress by regulating the inflammatory mediators and antioxidant enzymes in vivo.

### 3.5. STE Promoted the Production of Fecal SCFAs

SCFAs are a class of important signaling molecules associated with intestinal barrier function and colon inflammation [1]. Thus, we further tested by GC–MS whether STE intervention could change the composition and content of fecal SCFAs. As shown in Table 2, exposure to DSS without other treatment obviously decreased the contents of isobutyric acid, butyric acid, valeric acid, and caproic acid, while it significantly increased the contents of acetic acid and propionic acid (*p* < 0.05) in the MC group. However, the changes in acetic acid and butyric acid were significantly blocked by STE intervention (vs. the MC group; *p* < 0.05), especially butyric acid, which was increased by 74.80% and 93.36% in the L-STE and H-STE groups, respectively.

### 3.6. STE Regulated Butyric-Acid-Mediated GPRs and HDAC3/NF-κB Signaling

**Figure 5:**
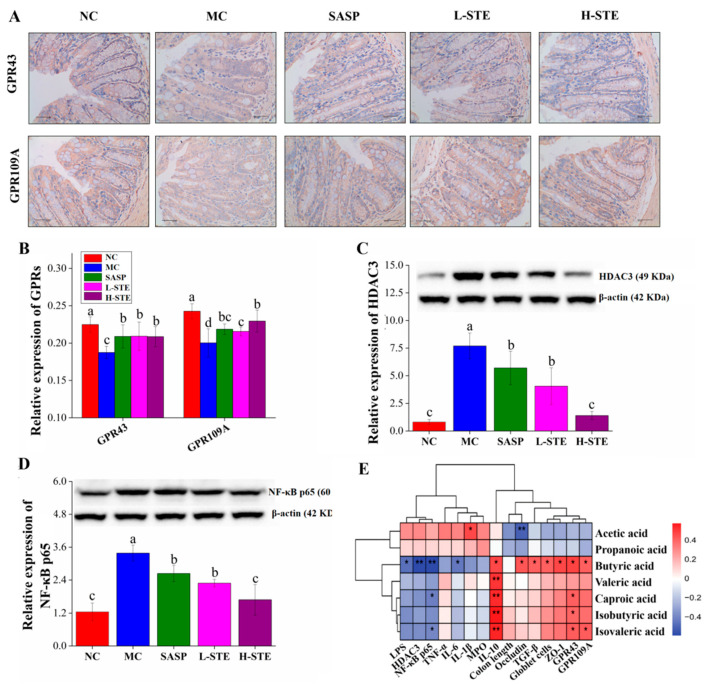
Effects of STE on the expression of GPRs, HDAC3, and NF-κB p65 in the colon of DSS-induced ulcerative colitis mice. (**A**) The immunohistochemistry analysis of GPR43 and GPR109A in the colon (magnification of 400×). (**B**) The expression of GPR43 and GPR109A in the colon. (**C**) The expression of HDAC3 protein in the colon. (**D**) The expression of NF-κB p65 protein in the colon. (**E**) Correlation between SCFAs and indicators of intestinal barrier and inflammatory indexes (* *p* < 0.05, and ** *p* < 0.01). Values are expressed as the mean ± the SD (*n* = 5). Bars with different lowercase letters indicate significant differences (*p* < 0.05).

SCFAs have been reported to activate GPR signaling, thereby regulating the production of inflammation-related cytokines [4]. Since butyric acid was significantly increased by STE treatment, we further investigated whether STE intervention could regulate the main receptors of butyrate, GPR43, and GPR109A. Immunohistochemical staining was first used to determine the expression of target proteins GPR43 and GPR109A in the colon. As shown in Figure 5A,B, the expression levels of GPR43 and GPR109A in the MC group were obviously reduced by 16.64% and 17.48%, respectively, compared to the NC group (*p* < 0.05; Figure 5B). However, STE treatment dramatically up-regulated the expression of GPR43 and GPR109A, which was consistent with the effect of the positive control SASP (Figure 5B).

Given that butyric acid is related to the HDAC3/NF-κB inflammatory signaling pathway [4,7], the expression of HDAC3 and NF-κB p65 was further investigated by WB. As shown in Figure 5C,D, the levels of the proteins HDAC3 and NF-κB p65 in the MC group were 8.49-fold and 1.72-fold higher than those in the NC group, respectively. However, the above changes were significantly blocked on treatment with STE (vs. the MC group; *p* < 0.05). Especially in the H-STE group, the levels of the proteins HDAC3 and NF-κB p65 were reduced by 81.89% and 50.32%, respectively (Figure 5C,D), with the effect better than the effect on SASP intervention. Together, these results suggest that STE might activate GPR43 and GPR109A signaling and inhibit HDAC3/ NF-κB signaling to improve UC.

### 3.7. STE Altered the Gut Microbiota Structure

**Figure 6:**
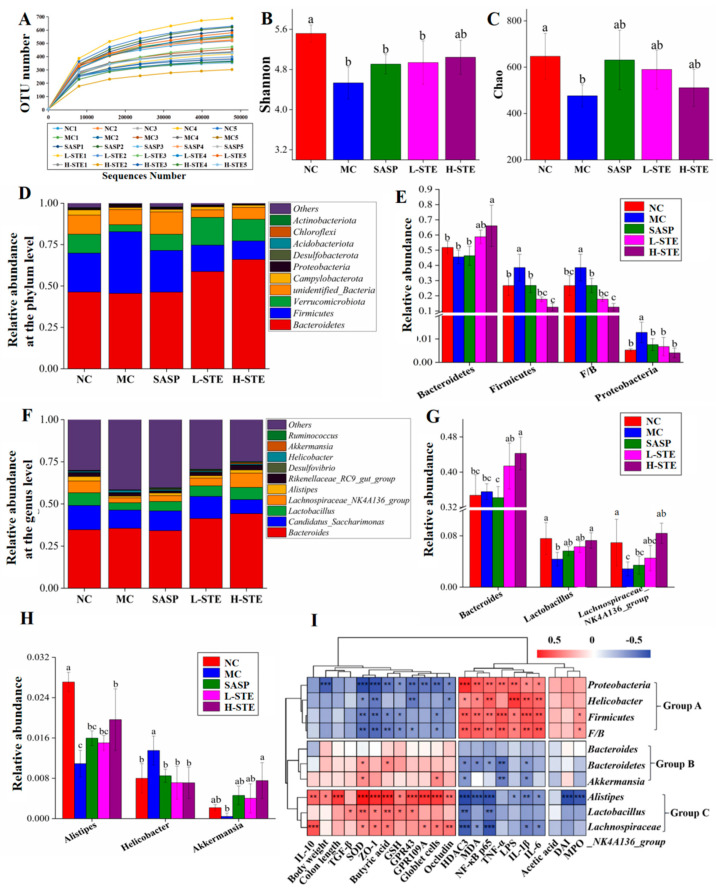
Effects of STE on the composition and abundance of gut microbiota in DSS-induced ulcerative colitis mice. (**A**) The number of observed OTUs. (**B**) Shannon index. (**C**) Chao index. (**D**) Distribution of gut microbiota at the phylum level. (**E**) Intestinal microbiota with significant differences at the phylum level. (**F**) Distribution of gut microbiota at the genus level. (**G**,**H**) Intestinal microbiota with significant differences at the genus level. (**I**) Correlation between the significantly changed gut microbiota and biochemical parameters (* *p* < 0.05, ** *p* < 0.01, and *** *p* < 0.001). Data are expressed as the mean ± the SD (*n* = 5). Bars with different lowercase letters indicate significant differences (*p* < 0.05).

Studies increasingly indicate that gut microbiota play a critical role in the etiology of UC [1]. We, thus, next used 16S rRNA gene sequencing to investigate whether STE could regulate the gut microbiota in DSS-induced UC mice. A total of 3,918,991 original 16S rRNA reads were obtained from 25 fecal samples (*n* = 5 per group) and further divided into 504 distinct operational taxonomic units (OTUs). As depicted in Figure 6A, the sequencing depth of this study was sufficient based on the OTU curve, which tended to be flat. In addition, according to the alpha diversity analysis, DSS treatment obviously reduced the Shannon and Chao indexes in the MC group (vs. the NC group; *p* < 0.05), but STE or SASP intervention overall did not significantly alter the above changes (Figure 6B,C; *p* > 0.05). Moreover, taxonomic characteristics of each group at the phylum and genus levels were compared to evaluate specific changes in the intestinal flora. First, a total of 10 different phyla were detected, and *Bacteroidetes* and *Firmicutes* were found as the leading phyla, accounting for more than 73% of the total abundance in all groups (Figure 6D). In addition, the MC group showed significant increases in the relative abundance of *Firmicutes* (38.54% vs. 26.67%) and *Proteobacteria* (1.28% vs. 0.52%) and the *Firmicutes*/*Bacteroidetes* (*F*/*B*) ratio (0.39% vs. 0.27%) when compared with the NC group (Figure 6E; *p* < 0.05). However, H-STE treatment significantly blocked these changes and increased the relative abundance of *Bacteroidetes* by 44.91% (*p* < 0.05) compared to the MC group (Figure 6E). Furthermore, the taxonomic communities at the genus level are shown in Figure 6F. Compared with the MC group, H-STE intervention significantly increased the abundance of *Lactobacillus* (Figure 6G), *Lachnospiraceae*_NK4A136_group (Figure 6G), and *Alistipes* (Figure 6H) and also blocked the increased relative abundance of *Helicobacter* in DSS-induced UC mice (Figure 6H). In particular, compared with the NC group, the abundance of *Bacteroides* (Figure 6G) and *Akkermansia* (Figure 6H) did not significantly change in the MC group but significantly increased in the H-STE group (Figure 6; *p* < 0.05). These results suggest that STE could alter the intestinal microbiota of DSS-induced UC mice.

### 3.8. Correlation Analysis

To further explore the possible intrinsic relationships among the contents of SCFAs, biochemical parameters, and gut microbiota composition, Spearman’s correlation analysis was conducted. As the heatmap shows in Figure 5E, the content of acetic acid was positively correlated with the level of IL-1β but negatively correlated with the expression level of Occludin (*p* < 0.05 or *p* < 0.01), suggesting that it may promote colon inflammation and have adverse effects on the intestinal barrier function. Nevertheless, butyric acid, valeric acid, isobutyric acid, and isovaleric acid showed the opposite effect to acetic acid. In particular, the content of butyric acid was positively correlated with IL-10; TGF-β; the number of goblet cells; and the levels of ZO-1, Occludin, GPR43, and GPR109A but negatively correlated with the levels of LPS and IL-6 and the expression of HDAC3 and NF-κB p65 (*p* < 0.05 or *p* < 0.01), indicating the importance of butyric acid in improving intestinal barrier function and inhibiting the HDAC3/NF-κB inflammatory signaling cascade.

As shown in Figure 6I, the gut microbiota were clustered into three groups. On the whole, the group A flora significantly enriched in the DSS treatment group, including *Firmicutes*, *Proteobacteria*, and *Helicobacter*, as well as the ratio of *F*/*B*, were positively correlated with the levels of pro-inflammatory mediators (LPS, IL-1β, IL-6, TNF-α, MDA, and MPO) and the expression of HDAC3 and NF-κB p65, but negatively correlated with colon antioxidant indicators (GSH and SOD), the mucosal barrier function (the number of goblet cells and the expression of Occludin and ZO-1), butyric acid content, and levels of GPR43 and GPR109A. These results suggest that the proliferation of the above bacteria might destroy the intestinal barrier and induce colitis. Comparatively, the gut microbiota in group B, including *Bacteroidetes* and *Akkermansia*, were more negatively correlated with the pro-inflammatory mediators (IL-1β and TNF-α) and positively correlated with the colon antioxidant enzyme SOD, indicating that an increase in those bacteria might help improve the anti-inflammatory and antioxidant capacity of the colon. In addition, the gut microbiota in group C, including *Alistipes*, *Lactobacillus*, and *Lachnospiraceae*_NK4A136_group, mainly exhibited a positive correlation with the levels of IL-10, SOD, butyric acid, GPR43, and GPR109A, as well as the mucosal barrier function (the number of goblet cells and the expression of Occludin and ZO-1), but showed a negative correlation with the HDAC3/NF-κB inflammatory signal and related pro-inflammatory mediators (IL-1β and IL-6), suggesting that their proliferation might promote the production of butyric acid, restore mucosal barrier function, and inhibit inflammatory signals in UC mice.

## 4. Discussion

In the present study, the preventive effect and related mechanism of STE on DSS-induced UC in mice were investigated. We found that the intake of 3% DSS caused various disease symptoms similar to clinical colitis in *BABL*/c mice, including body weight loss, diarrhea complicated with hematochezia, shortened colon length, and increased DAI, which were considerably attenuated on treatment with STE. Moreover, STE supplementation significantly prevented DSS-induced colonic inflammation and oxidative stress and improved colon histopathological injury and intestinal epithelial barrier damage. These beneficial functions might be contributed to the activation of GPR43 and GPR109A signaling and the inhibition of HDAC3/NF-κB inflammatory signaling by regulating the composition and abundance of gut microbiota and promoting the production of butyric acid.

Colon histopathological injury and disruption of intestinal barrier function are considered to be the major hallmarks of UC [20]. It is well known that intestinal epithelial cells are coupled together through tight junction proteins (such as Occludin and ZO-1) to form a tight physical barrier that isolates the host’s innate immune system from intestinal microbiota [16]. However, inflammatory cell infiltration and tight junction protein loss in patients with UC can lead to the disruption of the intestinal epithelial barrier and further facilitate the translocation of LPS from the gut into the circulation system, thereby exacerbating the inflammatory response in a vicious cycle [21,22]. Therefore, the level of LPS in blood is considered to be a preliminary indicator of intestinal mechanical barrier damage [21,23]. In addition, HE and AB-PAS staining of colon tissue can provide visual evidence of histopathological changes in the intestinal mucosa. Similar to previous reports [1,7], DSS-induced UC mice presented significant impairment of the intestinal epithelial barrier, such as inflammatory cell infiltration, fibrous tissue hyperplasia, goblet cell depletion, and crypt structure loss. STE treatment obviously alleviated the above lesions, decreased serum LPS, and increased the expression of tight junction proteins Occludin and ZO-1 (Table 1; Figure 4). Therefore, as per these results, STE supplementation showed a protective effect on the intestinal barrier integrity in UC mice.

Immunological impairment and persistent inflammation mediated by cell–cytokine interactions are considered to be important contributors to intestinal barrier damage and the development of UC [24]. Neutrophils, macrophages, and dendritic cells are the main immune cells in the innate immune response to UC [11]. The regulation of these cells leads to the imbalance of inflammatory cytokines and then promotes the progress of local and systemic inflammation [16]. MPO is a representative pro-inflammatory and pro-oxidative enzyme, and its activity is regarded as a specific biomarker of neutrophil influx that causes mucosal damage in UC [25]. TNF-α is an inflammatory endogenous mediator mainly released by lymphocytes and macrophages, which can regulate the initiation and spread of inflammation [12,25]. IL-1β and IL-6 are two important pro-inflammatory factors positively correlated with colonic inflammation. Among them, IL-1β mainly mediates the sustained inflammatory response at the initial stage and IL-6 mainly acts on intestinal mucosal lesions [12,21]. IL-10 and TGF-β are two essential anti-inflammatory cytokines known to maintain intestinal immune homeostasis and have been proved to have protective effects against UC [16]. In our study, the levels of pro-inflammatory mediators, including MPO, TNF-α, IL-6, and IL-1β, were significantly increased after DSS exposure, while the levels of IL-10 and TGF-β decreased, indicating the occurrence of excessive inflammatory responses in the colon. However, STE treatment blocked these abnormalities in a dose-dependent manner (Table 1), which was consistent with the intervention of berberine hydrochloride [2] and lychee pulp phenolics [7]. In addition to inflammatory factors, oxidative stress is considered as an important factor involved in the occurrence and progress of intestinal inflammation [26]. Moreover, excessive inflammatory reaction can induce oxidative stress by stimulating the production of reactive oxygen/nitrogen species to exacerbate disease severity [12,26]. Therefore, we further examined three main markers of oxidative stress in the colon. The results showed that after STE treatment, the content of MDA, which is an indicator of oxidative damage, was significantly reduced in the colon tissue, while the levels of GSH and SOD, two important endogenous antioxidants against oxidative stress [27], were significantly increased (Table 1). These results were consistent with previous studies [2,12,16], suggesting that STE intervention could balance intestinal inflammatory factors and enhance antioxidant function in DSS-induced UC mice.

SCFAs are important metabolites in maintaining the homeostasis of the intestinal environment and regulating the host’s inflammatory response [28]. In particular, butyric acid, as a vital energy source for intestinal epithelial cells, has been widely proved to improve the defense of intestinal epithelial cells by increasing mucin and tight junctions [1,29]. In our study, STE notably increased the content of fecal butyric acid, which was positively related to the indicators of gut barrier integrity (e.g., goblet cells, ZO-1, and Occludin), suggesting that butyric acid might be a key factor for STE to improve the integrity of the intestinal barrier in UC mice (Table 2; Figure 5). In addition to improving the protective effect of the mucosal barrier, butyric acid is also considered to be a crucial substance in the intestinal anti-inflammatory response [30]. On the one hand, butyric acid acts as a ligand of metabolite-sensing GPRs, including GPR43 and GPR109A, on the colonic epithelial cells, to promote the generation of regulatory T cells and anti-inflammatory cytokines, such as TGF-β and IL-10 [30,31,32]. On the other hand, there is sufficient evidence supporting that butyric acid participates in inflammation regulation by acting as an inhibitor of HDACs, thus inactivating the downstream NF-κB pathway and down-regulating the production of colonic pro-inflammatory cytokines, such as TNF-α, IFN-γ, IL-2, and IL-6 [32,33]. Supplementation of lychee pulp phenolics was found to activate butyric acid–GPR43 signaling and suppress the TLR4/NLRP3-NF-κB pathway to alleviate DSS-induced intestinal inflammation [7]. In addition, the treatment of germinated barley foodstuff increased the fecal butyric acid content and inhibited NF-κB binding activity [34]. These results suggest the important role of butyric acid in controlling colitis via regulating the GPRs and NF-κB signaling. The present work found that STE intervention markedly promoted the expression of GPR43 and GPR109A and inhibited the expression of HDAC3 and NF-κB p65, which was significantly correlated with the butyric acid content (Figure 5). On account of those results, we speculated that butyric-acid-mediated activation of GPRs (GPR43 and GPR109A) signaling and the inhibition of the HDAC3/NF-κB inflammatory pathway might be one of the potential mechanisms of STE against UC.

Gut microbiota are also one of the important factors affecting the mucosal barrier function and the host immune system, which has been considered a new target for controlling UC [1]. *Bacteroidetes* is the most abundant group in the gut, which can interact with treg cells to promote the secretion of IL-10, thereby protecting mice from pathogen-induced colitis [15,35]. *Firmicutes*, a community of Gram-positive bacteria, are related to the impairment of gut barrier integrity and LPS leakage [12]. Thus, a high ratio of *F*/*B* is considered to be one important marker of gut microbiota dysbiosis [7]. In addition, *Proteobacteria* includes a variety of pathogenic bacteria, such as *Helicobacter* and *Enterohepatic*, which are positively related to intestinal mucosal injury and inflammatory diseases [7,36]. The available experimental and clinical studies have reported that compared with healthy individuals, UC patients have lower concentrations of *Bacteroidetes* and organic acids and their inflamed membranes show more *Firmicutes* and *Proteobacteria* [37]. In our study, gut microbiota dysbiosis in DSS-induced UC mice was also observed, such as the prominently increased abundance of *Firmicutes*, *Proteobacteria*, and *Helicobacter*, as well as a higher *F*/*B* ratio, which were positively correlated with the colonic inflammation, oxidative stress, and mucosal barrier damage. However, STE significantly reversed these changes and increased the abundance of *Bacteroidetes*, suggesting that STE might prevent intestinal barrier damage and colon inflammation by inhibiting the proliferation of these bacteria. STE intervention notably enriched the abundance of some beneficial bacteria, such as *Bacteroides*, *Lactobacillus*, *Akkermansia*, *Lachnospiraceae_NK4A136_group*, and *Alistipes*, among which *Lactobacillus*, *Lachnospiraceae_NK4A136_group*, and *Alistipes* were positively correlated with the content of IL-10, butyric acid, and SOD, as well as the levels of GPR43, GPR109A, ZO-1, and Occludin, but negatively correlated with the HDAC3/NF-κB inflammatory signals (Figure 6). *Lactobacillus* is a probiotic known to promote SCFA production and mucin secretion [15]. Similarly, *Akkermansia* has been reported to reduce intestinal permeability and improve colon inflammation by increasing the thickness of the mucus layer [38]. In addition, both *Lachnospiraceae_NK4A136_group and Alistipes* belong to SCFA-producing bacteria, especially related to the biosynthesis of butyric acid [39]. Therefore, we speculated that STE promoted the biosynthesis of butyric acid through the enrichment of the above beneficial bacteria, followed by activating the GPR (GPR43 and GPR109A) signaling and inhibiting the HDAC3/NF-κB inflammatory pathway.

In our study, trilobatin, phloridzin, phloretin, and isoquercitrin were found as the main bioactive components of STE. Shen et al. [40] demonstrated that trilobatin supplementation could significantly decrease the abundance of *Firmicutes* and *Proteobacteria* and increase the relative abundance of *Lactobacillus*, *Bacteroides*, and *Oscillospira*. In addition, phlorizin was reported to act in the intestine to change the structure of gut microbiota, increasing the abundance of SCFA-producing beneficial bacteria, such as *Akkermansia*, *Lactobacillus*, and *Prevotella* [41]. Wu et al. [16] also reported that phloretin treatment increased the levels of *Bacteroidetes*, *Alistipes*, and *Lactobacillus*, which were negatively correlated with the disease symptoms and colon inflammation in UC mice. Our results are generally in agreement with the changes in the microbial community reported in the above studies. Collectively, it was suggested that trilobatin, phloridzin, and phloretin might be the predominant contributors of STE in remodeling the gut microbiota structure of UC mice by enhancing the abundance of beneficial bacteria and butyric-acid-producing bacteria and inhibiting pathogenic bacteria.

**Figure 7:**
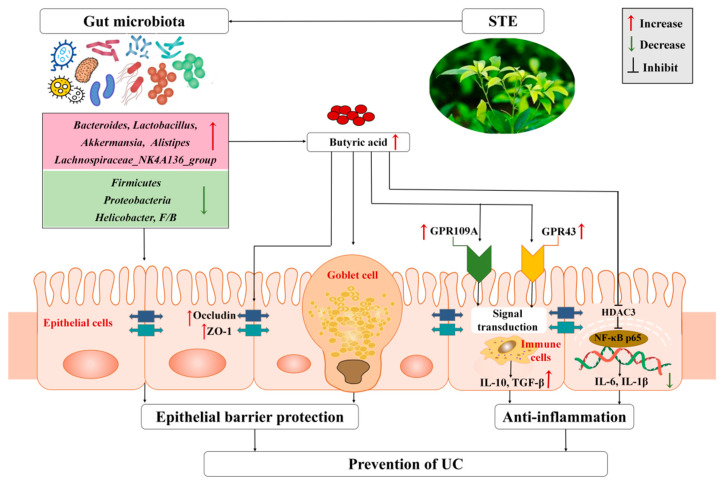
Mechanism of STE to attenuate DSS-induced ulcerative colitis through microbiota and their metabolites. ZO-1, zonula occludens-1; GPR109A, G-protein-coupled receptor 109A; GPR43, G-protein-coupled receptor 43; IL-10, interleukin-10; TGF-β, transforming growth factor-β; HDAC3, histone deacetylase 3; NF-κB p65, nuclear factor-κB p65; TNF-α, tumor necrosis factor-alpha; IL-6, interleukin-6; IL-1β, interleukin-1β; UC, ulcerative colitis; STE, sweet tea extract.

Taken together, we propose a possible mechanism of STE against UC based on the regulation of gut microbiota and their metabolites (Figure 7). Specifically, STE prevented gut microbiota dysbiosis by decreasing the abundance of *Firmicutes*, *Proteobacteria*, and *Helicobacter* and increasing the abundance of *Bacteroidetes*, *Lactobacillus*, *Akkermansia*, *Lachnospiraceae*_NK4A136_group, and *Alistipes*, accompanied by a significant increase in the content of butyric acid to protect intestinal barrier function, up-regulate the butyric acid–GPR signaling cascade, and inhibit the HDAC3/NF-κB inflammatory signaling pathway, which eventually blocked intestinal inflammation and oxidative stress injury of UC mice.

Finally, the current study has some limitations. First, it included a limited number of experimental animals (10 in each group), which might not be enough. Second, this study analyzed the gut microbiota profile by 16S rRNA gene sequencing, with the sequencing depth not enough compared to metagenomics sequencing. Third, this study mainly explored the effect of STE on the prevention of UC, which, although important, cannot be directly extrapolated to clinical applications in UC patients. Therefore, it is still necessary to further investigate its therapeutic effect in animal models and humans in the future.

## 5. Conclusions

In conclusion, this study demonstrated that STE intervention could prevent DSS-induced UC symptoms, colon injury, intestinal inflammation, and oxidative stress and its potential mechanism might be related to the regulation of gut microbiota to activate the butyric acid–GPR (GPR43 and GPR109A) anti-inflammatory signaling cascade and simultaneously inhibit the HDAC3/NF-κB inflammatory signaling pathway. Therefore, sweet tea may be used as a healthy drink to reduce the risk of UC.

## Figures and Tables

**Table 1:** Effects of STE on the inflammatory and oxidative-related mediators.

Mediators	Groups				
	NC	MC	SASP	L-STE	H-STE
MPO (U/mgprot)	0.07 ± 0.03 ^c^	0.23 ± 0.10 ^a^	0.10 ± 0.05 ^bc^	0.15 ± 0.07 ^b^	0.12 ± 0.06 ^bc^
IL-6 (pg/mL)	2.31 ± 0.22 ^c^	3.30 ± 0.16 ^a^	2.62 ± 0.39 ^bc^	2.76 ± 0.40 ^b^	2.64 ± 0.30 ^bc^
IL-1β (pg/mL)	2.62 ± 0.14 ^c^	3.70 ± 0.37 ^a^	2.84 ± 0.51 ^bc^	2.74 ± 0.44 ^bc^	2.36 ± 0.27 ^b^
TNF-α (pg/mL)	30.44 ± 0.86 ^b^	38.49 ± 6.15 ^a^	29.29 ± 4.46 ^b^	28.31 ± 4.40 ^b^	25.97 ± 5.60 ^b^
LPS (pg/mL)	2.90 ± 0.44 ^c^	4.33 ± 0.36 ^a^	3.28 ± 0.30 ^bc^	3.31 ± 0.14 ^bc^	3.37 ± 0.38 ^b^
IL-10 (pg/mL)	20.34 ± 3.17 ^a^	14.16 ± 1.47 ^b^	17.45 ± 3.93 ^ab^	17.37 ± 3.42 ^ab^	18.20 ± 3.54 ^a^
TGF-β (pg/mL)	47.66 ± 1.36 ^a^	41.41 ± 1.60 ^c^	43.44 ± 1.81 ^bc^	44.94 ± 1.96 ^b^	45.14 ± 2.05 ^b^
SOD (U/mgprot)	843.42 ± 58.82 ^a^	640.74 ± 55.09 ^c^	737.87 ± 46.00 ^b^	738.59 ± 37.61 ^b^	767.09 ± 72.61 ^b^
GSH (mg/gprot)	3.90 ± 0.70 ^a^	1.26 ± 0.32 ^d^	1.71 ± 0.64 ^cd^	2.37 ± 0.96 ^bc^	2.98 ± 0.82 ^ab^
MDA (nmol/mgprot)	1.00 ± 0.14 ^d^	1.91 ± 0.22 ^a^	1.55 ± 0.22 ^b^	1.31 ± 0.26 ^bc^	1.25 ± 0.23 ^cd^

Abbreviations: MPO, myeloperoxidase; IL-6, interleukin-6; IL-1β, interleukin-1β; TNF-α, tumor necrosis factor-α; LPS, lipopolysaccharide; IL-10, interleukin-10; TGF-β, transforming growth factor-β; SOD, superoxide dismutase; GSH, glutathione; MDA, malondialdehyde; NC, normal control group; MC, DSS-induced UC model group; SASP, suffasalarin intervention group at the dose of 100 mg/kg; L-STE, STE intervention group at the dose of 100 mg/kg; H-STE, STE intervention group at the dose of 400 mg/kg. Data are expressed as the mean ± the SD (*n* = 10) and analyzed by one-way ANOVA followed by Duncan’s multiple comparisons test. The different superscript lowercase letters in the same column indicate statistical significance (*p* < 0.05).

**Table 2:** Effects of STE on the contents of main fecal SCFAs.

Groups	SCFAs (μg/g)						
	Acetic Acid	Propanoic Acid	Isobutyric Acid	Butyric Acid	Isovaleric Acid	Valeric Acid	Caproic Acid
NC	995.89 ± 80.17 ^b^	220.89 ± 21.03 ^b^	25.86 ± 1.64 ^a^	365.12 ± 88.77 ^a^	20.45 ± 2.16 ^a^	42.29 ± 1.87 ^a^	1.48 ± 0.51 ^a^
MC	1307.18 ± 101.68 ^a^	290.96 ± 34.29 ^a^	13.76 ± 0.40 ^b^	139.97 ± 23.48 ^b^	10.60 ± 0.28 ^b^	31.38 ± 1.48 ^b^	0.82 ± 0.17 ^b^
SASP	918.04 ± 137.65 ^b^	250.71 ± 25.97 ^ab^	16.44 ± 2.26 ^b^	179.87 ± 29.07 ^b^	12.29 ± 0.59 ^b^	27.73 ± 0.56 ^b^	0.80 ± 0.05 ^b^
L-STE	907.52 ± 171.62 ^b^	269.21 ± 12.53 ^a^	16.54 ± 1.62 ^b^	301.65 ± 22.48 ^a^	11.92 ± 1.08 ^b^	32.92 ± 2.22 ^b^	0.84 ± 0.03 ^b^
H-STE	879.19 ± 121.77 ^b^	286.76 ± 27.32 ^a^	14.91 ± 2.07 ^b^	315.01 ± 27.28 ^a^	11.63 ± 0.76 ^b^	29.25 ± 6.62 ^b^	0.84 ± 0.07 ^b^

Abbreviations: NC, normal control group; MC, DSS-induced UC model group; SASP, suffasalarin intervention group at the dose of 100 mg/kg; L-STE, STE intervention group at the dose of 100 mg/kg; H-STE, STE intervention group at the dose of 400 mg/kg. Data are expressed as the mean ± the SD (*n* = 10) and analyzed by one-way ANOVA followed by Duncan’s multiple comparisons test. The different superscript lowercase letters in the same column indicate statistical significance (*p* < 0.05).

## Data Availability

The data that support the findings of this study are openly available in [4TU. Research Data] at http://doi.org/10.4121/18865163, live link, https://figshare.com/s/3d6b12830753d741bf60 (accessed on 26 January 2022).

## Abbreviations

UC, ulcerative colitis; STE, sweet tea aqueous extract; DSS, dextran sulfate sodium; DAI, disease activity index; SASP, salicylazosulfapyridine; TNF-α, tumor necrosis factor-α; NF-κB p65, nuclear factor-κB p65; IL-1β, interleukin-1β; IL-6, interleukin-6; SCFAs, short-chain fatty acids; MPO, myeloperoxidase; GSH, glutathione; MDA, malondialdehyde; SOD, superoxide dismutase; TGF-β, transforming growth factor-β; IL-10, interleukin-10; LPS, lipopolysaccharide; ZO-1, zonula occludens-1; GPR43, G-protein-coupled receptor 43; GPR109A, G-protein-coupled receptor 109A; HDAC3, histone deacetylase 3; TPC, total phenolic content; TFC, total flavonoid content; NC, normal control group; MC, DSS-induced UC model group; SASP, suffasalarin (100 mg/kg) + DSS group; L-STE, low-dose sweet tea extract (100 mg/kg) + DSS group; H-STE, high-dose sweet tea extract (400 mg/kg) + DSS group; H&E, hematoxylin-eosin; AB-PAS, Alcian blue periodic acid Schiff; WB, Western blot; OTUs, operational taxonomic units; F/B, the *Firmicutes/Bacteroidetes* ratio.
